# Supplementary material for: A Statistical Analysis on the Effect of Antioxidants on the Thermal-Oxidative Stability of Commercial Mass- and Emulsion-Polymerized ABS
Source: Polymers (Basel). 2018 Dec 25;11(1):25. doi: 10.3390/polym11010025 (PMC6401883; doi:10.3390/polym11010025)

ANOVA Results: R1

Run	A	B	C	D	Replications (blocks)	
					1st	2nd
1	-	-	-	-	1.03	0.77
2	+	-	-	-	0.98	0.97
3	-	+	-	-	0.99	1.02
4	+	+	-	-	1.09	0.69
5	-	-	+	-	0.98	1.12
6	+	-	+	-	0.99	0.90
7	-	+	+	-	1.06	0.89
8	+	+	+	-	0.87	0.86
9	-	-	-	+	1.28	1.27
10	+	-	-	+	1.40	0.89
11	-	+	-	+	1.45	1.40
12	+	+	-	+	1.29	1.27
13	-	-	+	+	1.34	0.94
14	+	-	+	+	1.26	1.33
15	-	+	+	+	1.23	1.12
16	+	+	+	+	1.52	1.13

Alfa: 0.05

Source of Variation	SS	DF	MS	F0	P-Value	F Tab
Irganox 1076 (A)	0.00607	1	0.0060717445	0.3087990959	0.58661	4.54308
Irganox 245 (B)	0.00535	1	0.0053532791	0.27225911	0.60944	4.54308
AB	0.00609	1	0.0060888371	0.3096683991	0.58609	4.54308
Irgafos 168 (C)	0.00228	1	0.002280657	0.1159905205	0.73815	4.54308
AC	0.02122	1	0.0212216052	1.0792964824	0.31531	4.54308
BC	0.01985	1	0.0198547646	1.0097811861	0.33089	4.54308
ABC	0.00485	1	0.0048494014	0.2466327047	0.62666	4.54308
Type of ABS (D)	0.75943	1	0.7594264472	38.6231996656	0.00002	4.54308
AD	0.00984	1	0.0098381871	0.5003542703	0.49019	4.54308
BD	0.02873	1	0.0287334385	1.4613361657	0.24543	4.54308
ABD	0.00338	1	0.0033782145	0.1718105206	0.68437	4.54308
CD	0.00865	1	0.0086536879	0.4401125573	0.51713	4.54308
ACD	0.07118	1	0.0711767018	3.6199318231	0.07647	4.54308
BCD	0.00021	1	0.0002124376	0.0108042313	0.91859	4.54308
ABCD	0.00305	1	0.0030496098	0.155098215	0.69925	4.54308
Blocks	0.14916	1	0.1491603926	7.5860560905	0.01476	4.54308
Error	0.29494	15	0.0196624426			
Total	1.39429	31				

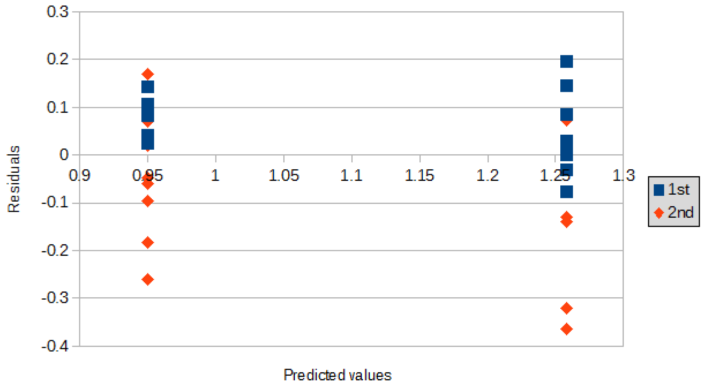

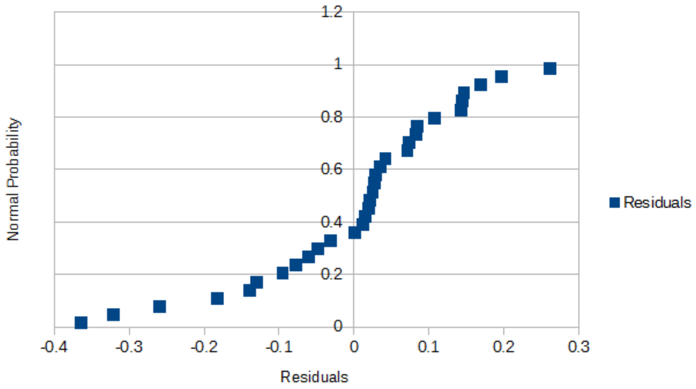

ANOVA Results: R2

Run	A	B	C	D	Replications (blocks) 1st	2nd
1	-	-	-	-	1.87	1.20
2	+	-	-	-	1.60	1.65
3	-	+	-	-	1.60	1.63
4	+	+	-	-	1.70	1.19
5	-	-	+	-	1.51	1.89
6	+	-	+	-	1.60	1.51
7	-	+	+	-	1.80	1.57
8	+	+	+	-	1.52	1.48
9	-	-	-	+	1.71	1.84
10	+	-	-	+	2.05	1.35
11	-	+	-	+	2.18	2.05
12	+	+	-	+	2.05	1.87
13	-	-	+	+	2.05	1.31
14	+	-	+	+	1.83	2.06
15	-	+	+	+	1.74	1.62
16	+	+	+	+	2.35	1.72

Alfa: 0.05

Source of Variation	SS	DF	MS	F0	P-Value	F Tab
Irganox 1076 (A)	2.5E-05	1	2.499245E-05	0.0004088974	0.98413	4.54308
Irganox 245 (B)	0.03307	1	0.0330656328	0.5409814143	0.47337	4.54308
AB	0.01172	1	0.0117167432	0.1916957206	0.66775	4.54308
Irgafos 168 (C)	8.1E-06	1	8.0802E-06	0.0001321988	0.99098	4.54308
AC	0.04419	1	0.044193645	0.7230450033	0.40852	4.54308
BC	0.03046	1	0.0304649928	0.4984327683	0.49101	4.54308
ABC	0.01842	1	0.0184204818	0.3013744923	0.59110	4.54308
Type of ABS (D)	0.62053	1	0.6205314205	10.1524131565	0.00613	4.54308
AD	0.07896	1	0.0789594061	1.291841938	0.27354	4.54308
BD	0.09175	1	0.0917504285	1.5011137651	0.23939	4.54308
ABD	0.01155	1	0.0115504801	0.1889755164	0.66996	4.54308
CD	0.02288	1	0.0228808832	0.3743503906	0.54980	4.54308
ACD	0.15025	1	0.1502506562	2.4582264306	0.13776	4.54308
BCD	0.03368	1	0.0336779105	0.5509987889	0.46938	4.54308
ABCD	0.00022	1	0.000222816	0.003645457	0.95265	4.54308
Blocks	0.32022	1	0.3202240392	5.2391331711	0.03701	4.54308
Error	0.91682	15	0.0611215689			
Total	2.38477	31				

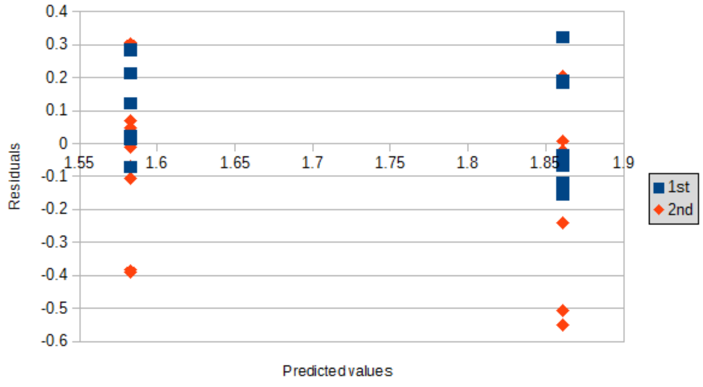

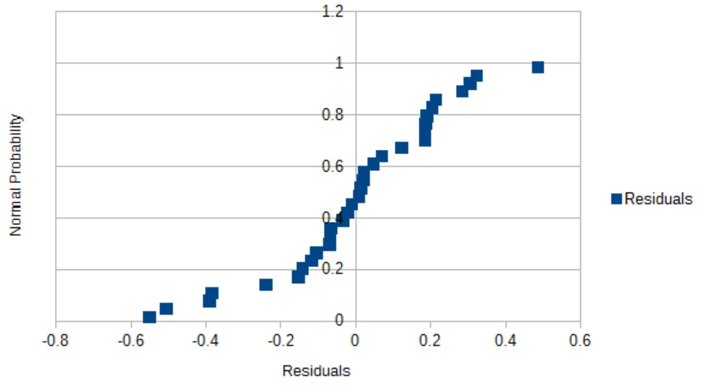

ANOVA Results: R3

Run	A	B	C	D	Replications (blocks) 1st	Replications (blocks) 2nd
1	-	-	-	-	1.14	1.41
2	+	-	-	-	1.26	1.35
3	-	+	-	-	1.39	1.42
4	+	+	-	-	1.31	1.21
5	-	-	+	-	1.36	1.45
6	+	-	+	-	1.25	1.37
7	-	+	+	-	1.29	1.32
8	+	+	+	-	1.31	1.37
9	-	-	-	+	1.83	1.98
10	+	-	-	+	1.79	2.03
11	-	+	-	+	1.96	2.10
12	+	+	-	+	1.80	2.11
13	-	-	+	+	1.52	1.68
14	+	-	+	+	1.68	2.18
15	-	+	+	+	1.77	2.08
16	+	+	+	+	2.09	2.10

Alfa: 0.05

Source of Variation	SS	DF	MS	F0	P-Value	F Tab
Irganox 1076 (A)	0.0072	1	0.0072006	0.6635536142	0.42805	4.54308
Irganox 245 (B)	0.0548	1	0.0547970513	5.0496877121	0.04011	4.54308
AB	0.01026	1	0.0102638628	0.9458410761	0.34620	4.54308
Irgafos 168 (C)	0.00228	1	0.0022767752	0.2098106283	0.65348	4.54308
AC	0.04992	1	0.0499201003	4.6002642731	0.04875	4.54308
BC	0.00322	1	0.003224045	0.2971039508	0.59372	4.54308
ABC	0.00647	1	0.006474651	0.5966555665	0.45186	4.54308
Type of ABS (D)	2.80704	1	2.807040027	258.6758814296	0.00000	4.54308
AD	0.04672	1	0.0467170745	4.3050972893	0.05562	4.54308
BD	0.05277	1	0.0527686341	4.8627639113	0.04346	4.54308
ABD	0.0047	1	0.0046977125	0.4329061563	0.52054	4.54308
CD	0.01635	1	0.016345224	1.5062539857	0.23863	4.54308
ACD	0.03477	1	0.034768845	3.2040375414	0.09366	4.54308
BCD	0.02818	1	0.0281829411	2.5971297393	0.12789	4.54308
ABCD	0.02009	1	0.0200880968	1.8511692373	0.19374	4.54308
Blocks	0.17816	1	0.1781627125	16.4181473144	0.00104	4.54308
Error	0.16277	15	0.0108515723			
Total	3.4857	31				

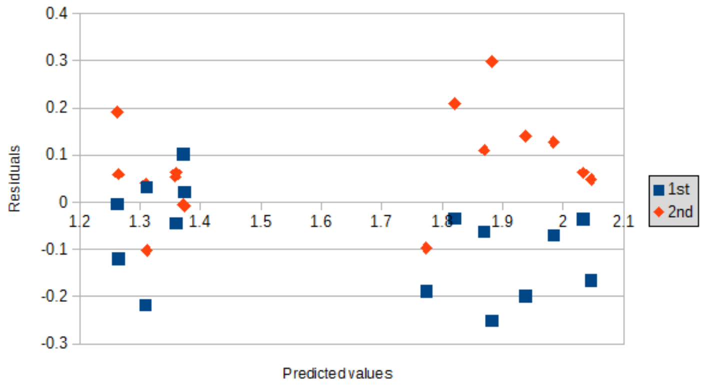

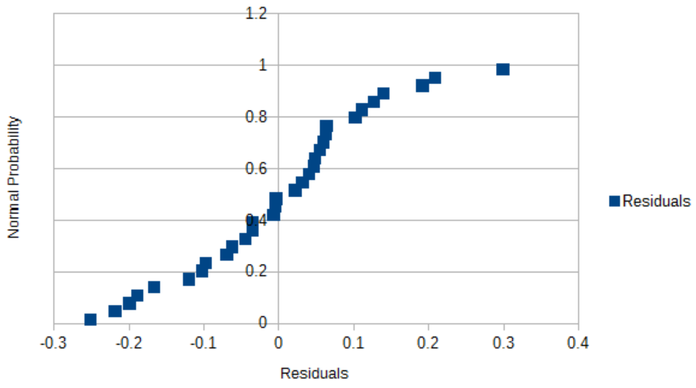

ANOVA Results: R4

Run	A	B	C	D	Replications (blocks) 1st	2nd
1	-	-	-	-	2.06	2.20
2	+	-	-	-	2.07	2.30
3	-	+	-	-	2.26	2.27
4	+	+	-	-	2.04	2.09
5	-	-	+	-	2.11	2.45
6	+	-	+	-	2.02	2.30
7	-	+	+	-	2.18	2.34
8	+	+	+	-	2.27	2.36
9	-	-	-	+	2.45	2.86
10	+	-	-	+	2.61	3.07
11	-	+	-	+	2.95	3.06
12	+	+	-	+	2.85	3.11
13	-	-	+	+	2.32	2.34
14	+	-	+	+	2.44	3.38
15	-	+	+	+	2.52	3.01
16	+	+	+	+	3.23	3.19

Alfa: 0.05

Source of Variation	SS	DF	MS	F0	P-Value	F Tab
Irganox 1076 (A)	0.11815	1	0.1181538966	3.8630751442	0.06815	4.54308
Irganox 245 (B)	0.23442	1	0.2344207392	7.664452522	0.01434	4.54308
AB	0.02101	1	0.0210084002	0.6868756	0.42022	4.54308
Irgafos 168 (C)	0.00157	1	0.0015663204	0.0512112911	0.82402	4.54308
AC	0.1136	1	0.1136023778	3.7142619463	0.07311	4.54308
BC	0.01634	1	0.016343416	0.5343526196	0.47605	4.54308
ABC	0.03231	1	0.0323050071	1.0562213654	0.32037	4.54308
Type of ABS (D)	3.16564	1	3.165643801	103.5016214746	0.00000	4.54308
AD	0.24876	1	0.248762838	8.1333715084	0.01212	4.54308
BD	0.1421	1	0.1421031361	4.6461023167	0.04778	4.54308
ABD	0.00854	1	0.0085360178	0.2790875218	0.60503	4.54308
CD	0.05115	1	0.0511488128	1.6723249342	0.21551	4.54308
ACD	0.07551	1	0.0755127522	2.4689108396	0.13697	4.54308
BCD	0.00175	1	0.0017478828	0.057147524	0.81430	4.54308
ABCD	0.01615	1	0.0161451465	0.5278701421	0.47869	4.54308
Blocks	0.48316	1	0.4831641602	15.7971891857	0.00122	4.54308
Error	0.45878	15	0.0305854513			
Total	5.18895	31				

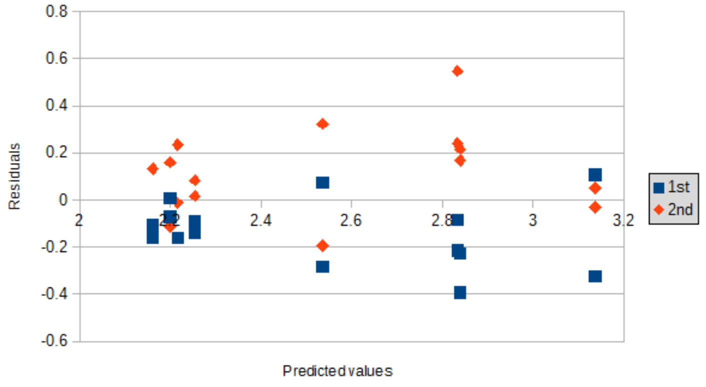

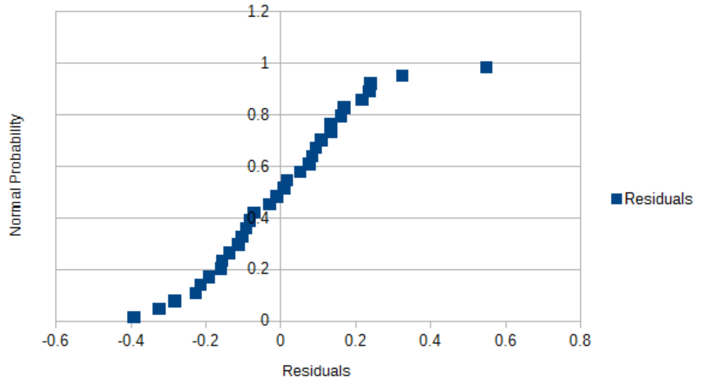

ANOVA Results: OOT

Run	A	B	C	D	Replications (blocks) 1st	2nd
1	-	-	-	-	160.20	182.50
2	+	-	-	-	202.70	191.60
3	-	+	-	-	188.30	203.03
4	+	+	-	-	196.50	193.20
5	-	-	+	-	177.30	194.45
6	+	-	+	-	197.80	198.00
7	-	+	+	-	197.60	196.20
8	+	+	+	-	200.20	207.90
9	-	-	-	+	159.40	166.50
10	+	-	-	+	195.90	194.40
11	-	+	-	+	179.50	187.10
12	+	+	-	+	195.60	196.10
13	-	-	+	+	172.40	172.40
14	+	-	+	+	187.60	195.20
15	-	+	+	+	181.20	188.10
16	+	+	+	+	202.10	193.00

Alfa: 0.05

Source of Variation	SS	DF	MS	F0	P-Value	F Tab
Irganox 1076 (A)	1824.38	1	1824.3820125	44.3002427929	0.00001	4.54308
Irganox 245 (B)	773.031	1	773.0312	18.7709973086	0.00059	4.54308
AB	409.552	1	409.5522	9.9448809362	0.00656	4.54308
Irgafos 168 (C)	148.436	1	148.43645	3.604382596	0.07704	4.54308
AC	43.5244	1	43.52445	1.0568749797	0.32022	4.54308
BC	7.01251	1	7.0125125	0.17028013	0.68570	4.54308
ABC	155.673	1	155.6730125	3.7801031817	0.07086	4.54308
Type of ABS (D)	457.38	1	457.3800125	11.1062515765	0.00454	4.54308
AD	131.95	1	131.9500125	3.2040535098	0.09366	4.54308
BD	0.00845	1	0.00845	0.0002051857	0.98876	4.54308
ABD	4.11845	1	4.11845	0.1000055546	0.75618	4.54308
CD	35.9552	1	35.9552	0.873075967	0.36490	4.54308
ACD	6.1952	1	6.1952	0.1504338797	0.70357	4.54308
BCD	0.59951	1	0.5995125	0.0145575593	0.90557	4.54308
ABCD	8.38451	1	8.3845125	0.2035954843	0.65829	4.54308
Blocks	133.58	1	133.5795125	3.2436215636	0.09184	4.54308
Error	617.733	15	41.1822125			
Total	4757.52	31				

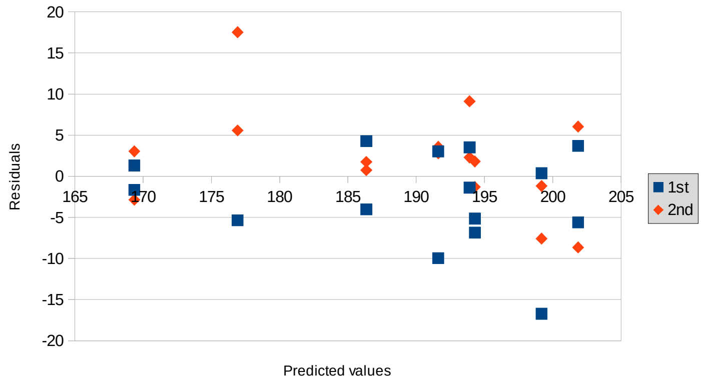

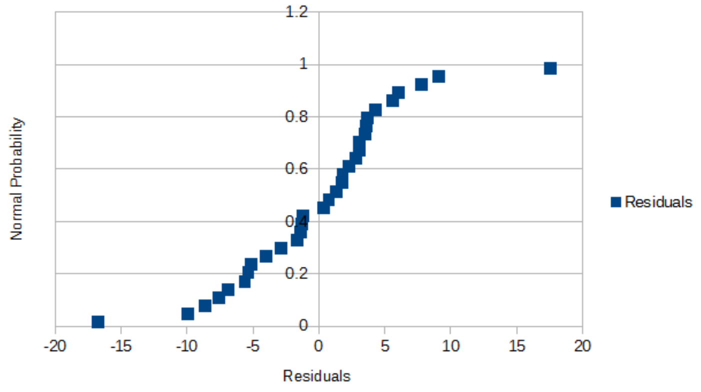

ANOVA Results: OP

Run	A	B	C	D	Replications (blocks) 1st	2nd
1	-	-	-	-	197.10	194.00
2	+	-	-	-	210.90	211.90
3	-	+	-	-	213.80	210.30
4	+	+	-	-	221.40	216.20
5	-	-	+	-	202.00	204.50
6	+	-	+	-	219.10	217.70
7	-	+	+	-	217.70	214.20
8	+	+	+	-	221.30	215.20
9	-	-	-	+	184.90	189.20
10	+	-	-	+	204.10	203.50
11	-	+	-	+	198.70	204.00
12	+	+	-	+	211.30	212.40
13	-	-	+	+	192.40	190.50
14	+	-	+	+	200.50	203.30
15	-	+	+	+	200.60	203.10
16	+	+	+	+	216.80	212.10

Alfa: 0.05

Source of Variation	SS	DF	MS	F0	P-Value	F Tab
Irganox 1076 (A)	1020.39	1	1020.3903125	162.2935574619	0.00000	4.54308
Irganox 245 (B)	835.383	1	835.3828125	132.8680278736	0.00000	4.54308
AB	84.8253	1	84.8253125	13.4915057109	0.00226	4.54308
Irgafos 168 (C)	69.9153	1	69.9153125	11.1200632226	0.00453	4.54308
AC	10.9278	1	10.9278125	1.7380737	0.20716	4.54308
BC	14.4453	1	14.4453125	2.2975337237	0.15037	4.54308
ABC	2.70281	1	2.7028125	0.4298835949	0.52198	4.54308
Type of ABS (D)	799	1	799.0003125	127.081374196	0.00000	4.54308
AD	13.1328	1	13.1328125	2.0887799835	0.16895	4.54308
BD	9.79031	1	9.7903125	1.5571537919	0.23121	4.54308
ABD	39.8278	1	39.8278125	6.3346322455	0.02370	4.54308
CD	19.3753	1	19.3753125	3.0816525344	0.09957	4.54308
ACD	0.11281	1	0.1128125	0.017942881	0.89522	4.54308
BCD	17.8503	1	17.8503125	2.8391005696	0.11268	4.54308
ABCD	18.4528	1	18.4528125	2.934928477	0.10727	4.54308
Blocks	3.44531	1	3.4453124998	0.5479785679	0.47057	4.54308
Error	94.3097	15	6.2873125			
Total	3053.89	31				

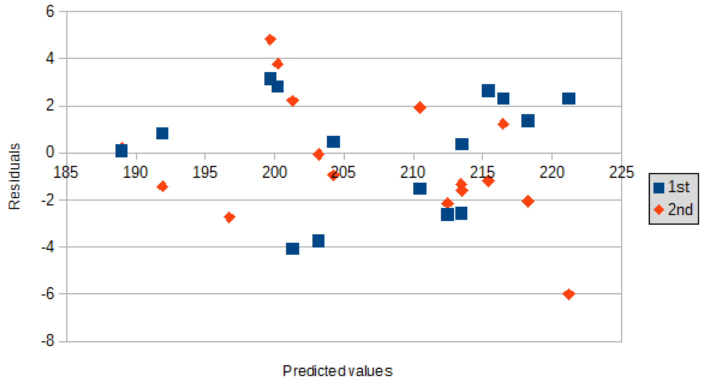

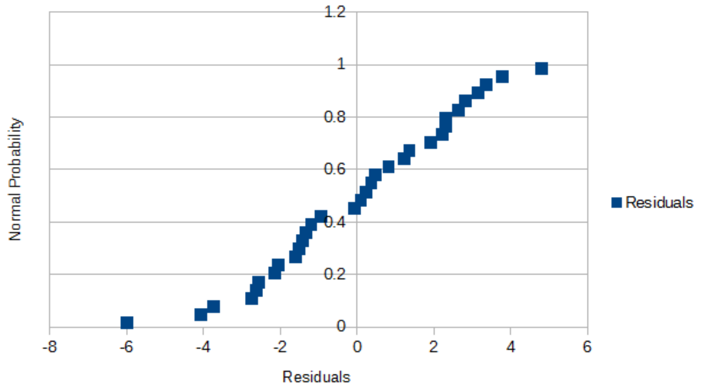

ANOVA Results: YI

Run	A	B	C	D	Replications (blocks) 1st	2nd
1	-	-	-	-	13.86	1.86
2	+	-	-	-	7.65	4.36
3	-	+	-	-	16.34	9.28
4	+	+	-	-	13.36	5.76
5	-	-	+	-	7.91	4.77
6	+	-	+	-	15.09	6.08
7	-	+	+	-	12.43	10.16
8	+	+	+	-	7.79	2.92
9	-	-	-	+	15.76	13.97
10	+	-	-	+	14.62	10.92
11	-	+	-	+	21.06	6.00
12	+	+	-	+	13.69	9.20
13	-	-	+	+	13.42	8.38
14	+	-	+	+	20.04	11.05
15	-	+	+	+	22.81	10.04
16	+	+	+	+	16.60	9.92

Alfa: 0.05

Source of Variation	SS	DF	MS	F0	P-Value	F Tab
Irganox 1076 (A)	11.2612	1	11.2612475247	1.4405045767	0.24867	4.54308
Irganox 245 (B)	9.70235	1	9.7023527261	1.2410954893	0.28279	4.54308
AB	46.891	1	46.8909547426	5.998148497	0.02709	4.54308
Irgafos 168 (C)	0.09443	1	0.0944257629	0.0120786568	0.91394	4.54308
AC	10.3027	1	10.3026619502	1.3178852218	0.26895	4.54308
BC	1.02934	1	1.0293357999	0.1316695089	0.72177	4.54308
ABC	34.5088	1	34.5088102277	4.414262182	0.05295	4.54308
Type of ABS (D)	189.461	1	189.4608222804	24.235252889	0.00018	4.54308
AD	2.08326	1	2.0832646911	0.2664848913	0.61323	4.54308
BD	7.31277	1	7.3127657262	0.9354267788	0.34879	4.54308
ABD	1.78762	1	1.7876221631	0.2286671971	0.63941	4.54308
CD	4.7773	1	4.7772995364	0.6110976454	0.44654	4.54308
ACD	0.63007	1	0.6300742163	0.0805971799	0.78037	4.54308
BCD	26.9171	1	26.9171124948	3.4431552682	0.08327	4.54308
ABCD	0.11551	1	0.1155131094	0.0147760861	0.90486	4.54308
Blocks	362.855	1	362.8547355135	46.4152755768	0.00001	4.54308
Error	117.264	15	7.8175715			
Total	826.993	31				

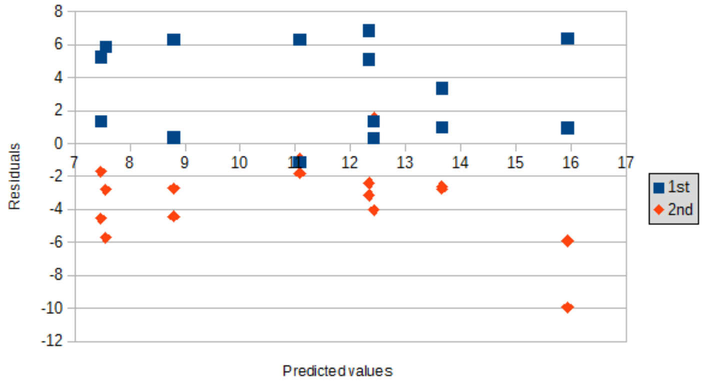

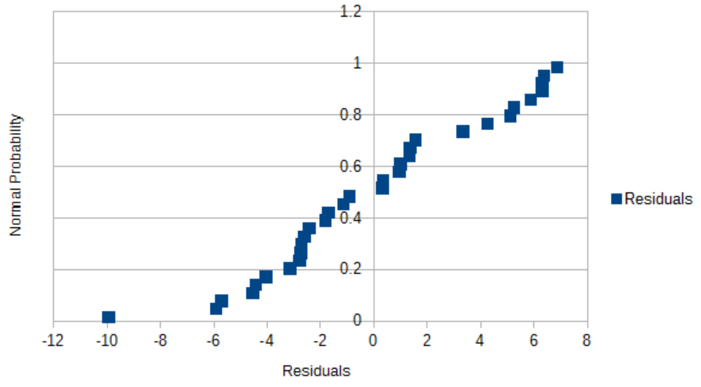

Supplement: Supplementary file 1 [file polymers-11-00025-s001.zip › polymers-405709-supplementary/Support Information - ANOVA Results.pdf]
