# Supplementary figures and images for: A Statistical Analysis on the Effect of Antioxidants on the Thermal-Oxidative Stability of Commercial Mass- and Emulsion-Polymerized ABS
Source: Polymers (Basel). 2018 Dec 25;11(1):25. doi: 10.3390/polym11010025 (PMC6401883; doi:10.3390/polym11010025)

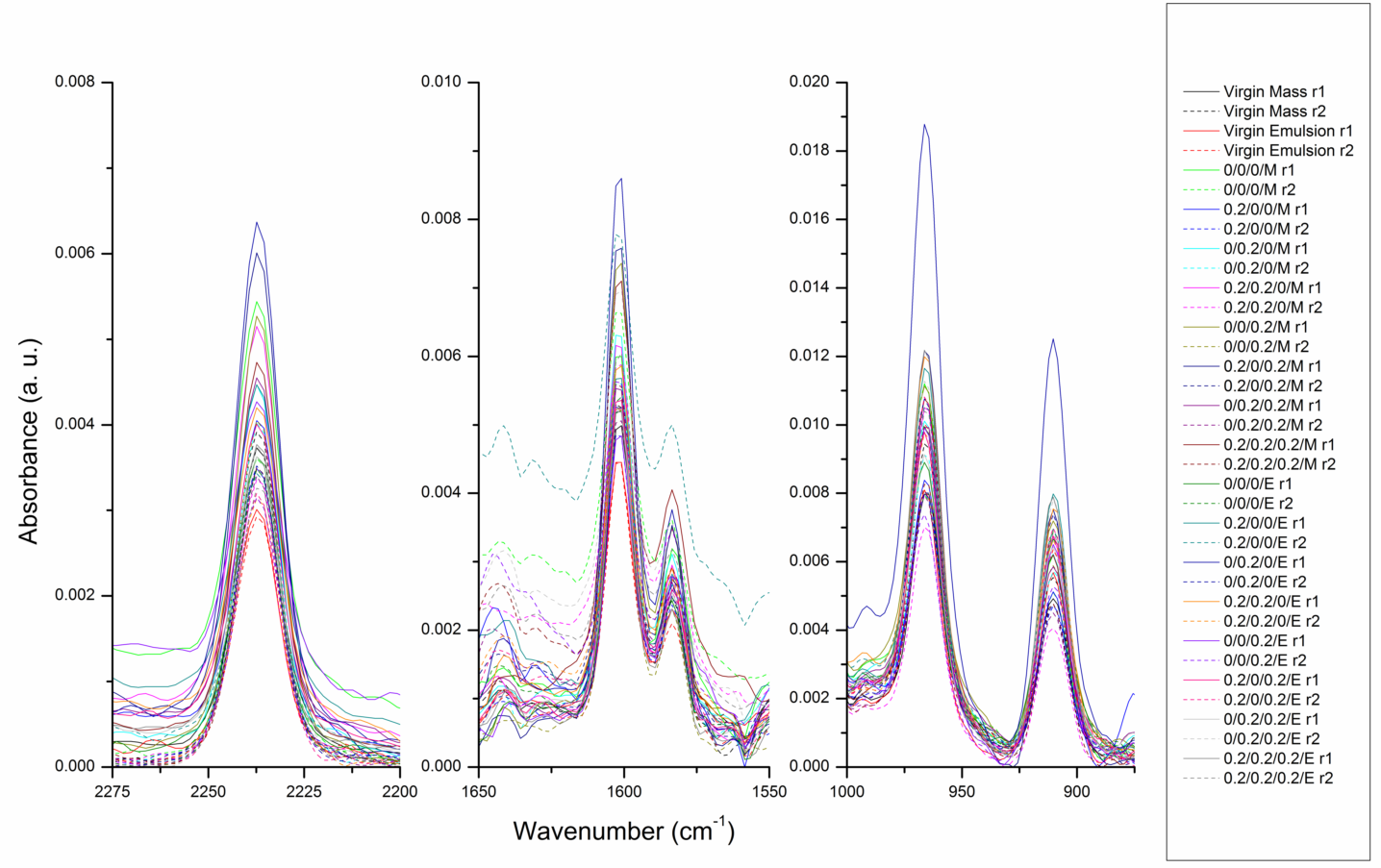


**Figure S1.** FTIR spectra.


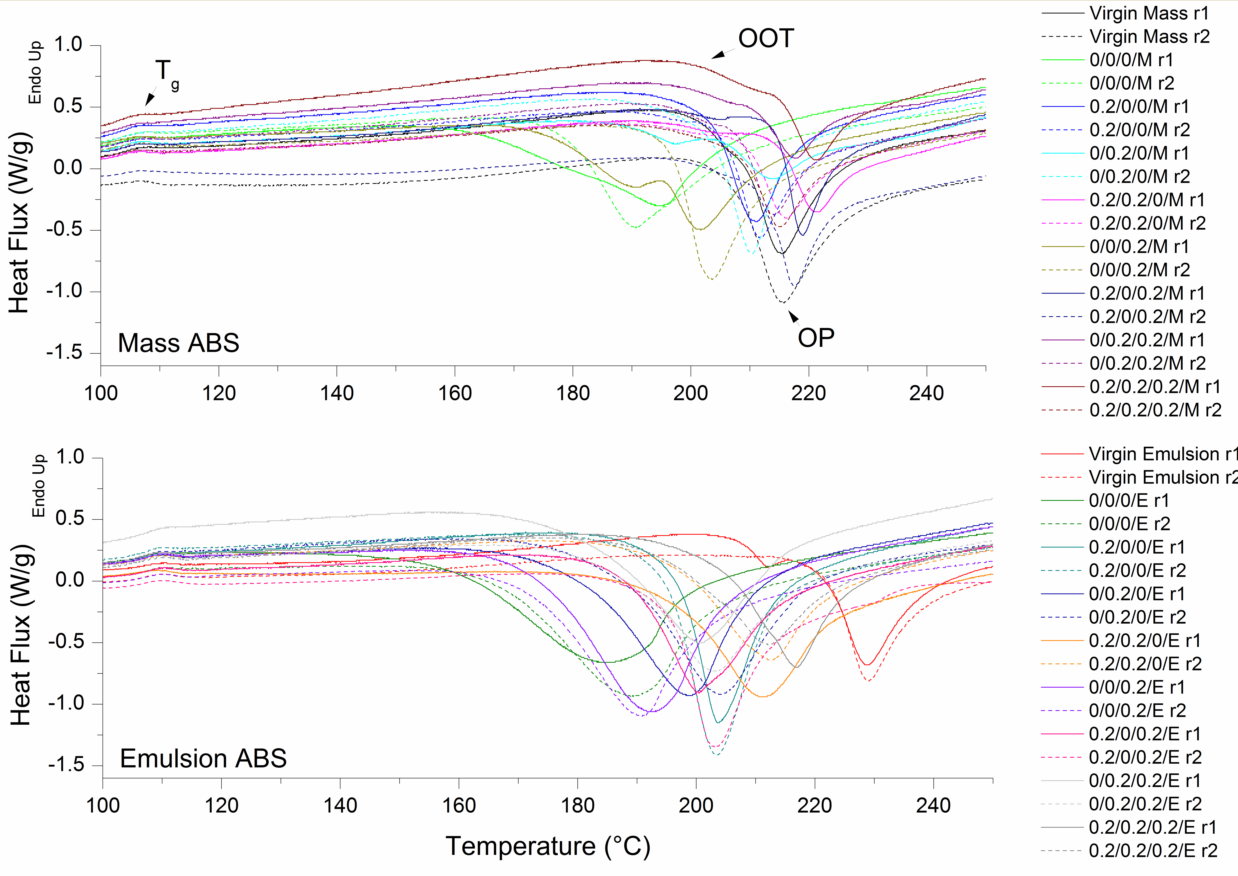


Figure S2: Oxidation onset temperature and oxidation peak.

Supplement: Supplementary file 1 [file polymers-11-00025-s001.zip › polymers-405709-supplementary/supplementary.docx]
